# A Regression Equation for the Estimation of Maximum Oxygen Uptake in Nepalese Adult Females

**DOI:** 10.5812/asjsm.34873

**Published:** 2010-03

**Authors:** Pinaki Chatterjee, Alok K Banerjee, Paulomi Das, Parimal Debnath

**Affiliations:** 1 Department of Physiology, SR College of Dental Sciences and Research, Faridabad, India; 2 Department of Physical Education, Kalyani University, Kalyani, West Bengal, India; 3 Department of Physiology, Nepalgunj Medical College, Chisapani, Banke, Nepal; 4 Department of Physical Education, Jadavpur University, Kolkata, India

**Keywords:** Cardiovascular fitness, VO_2_max, Beep test, Indirect measurement, Sedentary

## Abstract

**Purpose:**

Validity of the 20-meter multi stage shuttle run test (20-m MST) has not been studied in Nepalese population. The purpose of this study was to validate the applicability of the 20-m MST in Nepalese adult females.

**Methods:**

Forty female college students (age range, 20.42 ~24.75 years) from different colleges of Nepal were recruited for the study. Direct estimation of VO_2_ max comprised treadmill exercise followed by expired gas analysis by scholander micro-gas analyzer whereas VO_2_ max was indirectly predicted by the 20-m MST.

**Results:**

The difference between the mean (±SD) VO_2_ max values of direct measurement (VO_2_ max = 32.78 +/-2.88 ml/kg/min) and the 20-m MST (SPVO_2_ max = 32.53 + /-3.36 ml/kg/min) was statistically insignificant (*P*>0.1). Highly significant correlation (*r*=0.94, *P*<0.01) existed between the maximal speed of the 20-m MST and VO_2_ max. Limits of agreement analysis also suggest that the 20-m MST can be applied for the studied population.

**Conclusion:**

The results of limits of agreement analysis suggest that the application of the present form of the 20-m MST may be justified in the studied population. However, for better prediction of VO_2_ max, a new equation has been computed based on the present data to be used for female college students of Nepal.

## INTRODUCTION

Direct measurement of maximum oxygen uptake (VO_2_ max) is recognized as the best single index of aerobic fitness^[1]^. But the test of the direct measurement of cardiorespiratory endurance (VO_2_ max) itself is difficult, exhausting and often hazardous to perform regardless the type of ergometer used ^[2]^. Since the direct testing procedure is rather complicated on larger populations, several indirect running and walking field tests have been developed. Scientists often calculate VO_2_ max with indirect protocols^[3]^. It has been stated that equations for predicting VO_2_ max indirectly using field tests are very sensitive to populations tested on. Therefore, before applying any indirect protocol for prediction of VO_2_ max, the validity of the test should be established in a particular population. The 20-meter multistage shuttle run test (20-m MST); ^[4, 5]^, popularly known as Beep test, is often used worldwide for measurement of aerobic capacity ^[6,7,8,9,10]^. But in Nepal, the scientists have not yet used this test. Cooper et al,^[11]^ studied the repeatability and criterion related validity of the 20-m multistage fitness test as a predictor of maximal oxygen uptake in active young men. Suminski et al,^[12]^ established the validity of the 20-m MST for measuring aerobic fitness of Hispanic youth of 10 to 12 years of age. Chatterjee et al, ^[13, 14]^ studied the validity of 20-m MST in junior Taekwondo players and male university students of India. However, the validity and suitability of this test have not been studied in any Nepalese population until now. Nepal is the neighboring country of India, but a point to be noted here is that there are racial differences as well as differences in habitual activities and that the people of Nepal live at high altitudes.

A recent study suggests that gender-distinctive equations provide more accurate prediction of VO_2_ max from 20-m MST ^[15]^. For this reason, only female adults were recruited as subjects in the mentioned study and not males. Keeping in view all these facts, the present study was undertaken with an objective to assess the applicability of the 20-m MST to predict VO_2_ max in female college students of Nepal.

## METHODS AND SUBJECTS

**Subjects:** 40 female college students from different colleges of Nepal were volunteered for the study. The subjects had the mean age of 22.04 yr., height of 157.41 cm, and weight of 49.83 kg. The experimental protocol was fully explained to the participants and they underwent familiarization trial of the beep test few days before the actual test. They had a light breakfast 2-3 hours before the test and refrained from any energetic physical activity for that period. The participants had no history of any major disease and did not follow any physical-conditioning program, except from some recreational sports. The tests were demonstrated to the subjects before actual administration and they agreed to sign a statement of informed consent. All institutional policies concerning the human subjects in research were followed. The tests for all the subjects were done in the morning so that diurnal variation can be avoided, if there was any.

**Experimental Design:** Maximum oxygen consumption of each subject was determined by both indirect and direct methods at an interval of 4 days by random sequencing. Indirect one in the half of the subjects followed the direct method whereas indirect one was followed by direct method in the other half of the subjects. This was done so to avoid any possibility of bias. Subjects were asked to take complete rest at least for half an hour prior to the exercise, so that pulmonary ventilation and pulse rate might come down to a steady state ^[16]^.

**Prediction of maximum Oxygen uptake capacity by the 20-m MST:** Subjects started running back and forth a 20-m course and must touch the 20-m line. The initial speed was 8.5 km/hr. The speed got progressively faster (0.5 km/hr every minute), in accordance with a pace dictated by a sound signal on an audiotape. Several shuttle runs made up each stage. The subjects were instructed to keep pace with the signal for as long as possible. When the subjects could no longer follow the pace, the last stage announced was used to predict the maximal oxygen uptake using the equation of Leger et al.^[5]^. The equation:

Y=−27.4+6.0X,

Where Y = VO_2_ max (ml/kg/min) & X = Maximal shuttle run speed (km/hr)

**Direct measurement of maximum oxygen uptake capacity:** The subjects walked on a treadmill to warm up at a speed of 4 km/hr at a 4.5 inclination for five minutes ^[17]^. Running at a constant speed of 7 km/hr for a maximum duration of 5 min followed this. The inclination gradient was increased successively from 4.5 until the subject was unable to continue the task. In no case did it exceed 7.5 inclinations. The criteria to reach maximum state were exhaustion and withdrawal from running within the scheduled 5-min time period, when the heart rate reached the predicted maximum heart rate and when a further increase of inclination did not bring about any significant rise in oxygen uptake^[16]^.

Low resistance high velocity Collin's Triple “J type” plastic valve was used for the collection of gas by open circuit method^[16]^. The valve was connected with the Douglas bag (150-liter) and the expired gas was collected in the second minute of the exhausting final workload if signs of severe exhaustion supervened. No gas collection was made in the first minute of the workload. The expired gas measured in a wet gasometer (Toshniwal, Germany CAT No. C G 05.10) and the aliquots of gas samples were analyzed in a Scholander micro gas analysis apparatus following the standard procedure ^[18]^.

**Statistical Analyses:** The aired t-test, Pearson's product moment correlation, linear regression statistics and Bland and Altman approach for limit of agreement were adopted for statistical analyses of the data. Statistical package for Social Sciences (SPSS) MS windows Release 11.5 was used for statistical analyses.

To determine validity of the results, repeatability was investigated where 22 subjects performed the test (20-m MST) twice. The results showed non-significant bias between the two applications of the 20-m MST (mean of the difference +/− standard deviation of the difference = −0.13±1.8 ml/kg/min; *t =*−0.32; *P*=0.7 with 95% limits of agreement).

## RESULTS

Means and standard deviations of physical characteristics, predicted VO_2_ max (SPVO_2_ max) by 20-m MST and directly measured VO_2_ max of the participants are presented in the table 1.

**Table 1:** Physical parameters, predicted and measured VO_2_ max of the subjects (N=40)

Parameter	Minimum	Maximum	Mean	Std. Deviation
**Age (yr.)**	20.42	24.75	22.04	1.14
**Height (cm)**	154.10	160.30	157.41	1.79
**Weight (kg)**	42.50	57.20	49.83	4.21
**VO_2_max^‡^(ml/kg/min)**	26.90	38.00	32.78	2.88
SPVO_2_max* (ml/kg/min)	26.60	38.60	32.53	3.36
**Speed (km/hr)**	9.00	11.00	9.99	0.56

‡ VO_2_ max: maximum oxygen uptake

* SPVO_2_ max: predicted VO_2_ max

No significant variation was observed (*P*>0.1) between the values of directly measured and predicted VO_2_ max. The mean difference between VO_2_ max and SPVO_2_ max was 0.27 ml/kg/min with 95% confidence interval of −0.11 to 0.66 ml/kg/min. This indicates that 20-m MST predicted the maximum oxygen uptake capacity between −0.11 to 0.66 ml/kg/min. The standard error when predicting the VO_2_ max from shuttle run test was 0.53.

**Fig. 1:**
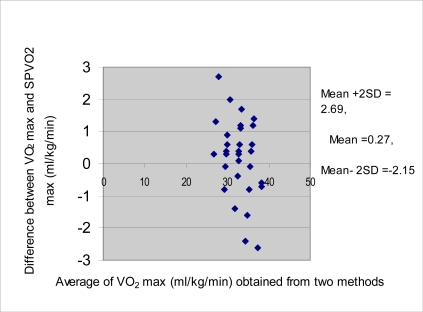
Plotting of difference between VO_2_ max values against their means(Bland and Altman method of approach)

Analysis of data by Bland and Altman^[19]^ method of approach for limits of agreement between SPVO_2_ max and VO_2_ max reveals that limits of agreement are –2.15 to 2.69 (Fig. 1). These parameters are small enough for the 20-m MST to be used confidently in place of the direct method. Limits of agreement analysis suggest that application of the present form of the 20-m MST should be justified for the studied population.

Highly significant correlation (*r*=0.94, *P*<0.01) existed between the maximal speed of the 20-m MST and VO_2_ max.

## DISCUSSION

The following equation, derived on the basis of present data will better predict the aerobic fitness in female college students of Nepal:

Y=−15.207+4.806 X

Where Y = VO_2_ max (ml/kg/min) and

X= Maximal shuttle run speed (km/hr)

Using the above new equation the limits of agreement between directly measured VO_2_ max and predicted VO_2_ max from the 20-m MST (SPVO_2_ max) are -2.01 to 2.03. The result suggests that better limits of agreement exist between the two methods when this newly developed equation is used for prediction of VO_2_ max from the 20-m MST.

Therefore, from the present observations it is concluded that the 20-m MST is recommended as a valid method to evaluate aerobic fitness in terms of VO_2_ max among female adults (age 20.42~24.75 yr.) of Nepal.

A recent study has indicated that there are sport-specific differences when predicting VO_2_ max results yielded from the MST ^[20]^. In another recent study by Cetin et al. on Taekwondo athletes, the authors concluded that VO_2_ max can be predicted from shuttle run test scores, but not as indicated with the test package. In order to obtain the true scores, one must apply a regression equation^[21]^. Studies by Chatterjee et al. on two different population of India also suggested separate regression equations for prediction of VO_2_ max in a particular population^[13, 14]^. In our present study too, it is found that 20-m MST can be used in the studied population, but for better prediction a new regression equation has been derived.

## CONCLUSION

The regression equation developed on the basis of present data is recommended to be used for the population studied. This is likely to be the most useful method when a large number of subjects are to be evaluated without the help of a well-equipped laboratory, with fewer expenses and within a short period of time. In a country like Nepal where laboratory facilities for direct evaluation of aerobic fitness is scanty, this method may be of great importance. Efforts should be taken to validate the applicability of 20-m MST in different Nepalese population including various sports disciplines.
